# Neonatal Presentation of Severe Hemophilia A: An Original Case Report and a Literature Review

**DOI:** 10.3390/children11111352

**Published:** 2024-11-06

**Authors:** Erika Alboreto, Federico Pezzotta, Francesco Vinci, Andrea Calandrino, Laura Banov, Federica Mongelli, Paolo Massirio, Silvia Buratti, Andrea Moscatelli, Luca Antonio Ramenghi

**Affiliations:** 1 Department of Neuroscience, Rehabilitation, Ophthalmology, Genetics, Mother and Child Health, School of Medical and Pharmaceuticals, University of Genoa, 16132 Genoa, Italy; s5286238@studenti.unige.it (E.A.); s5296194@studenti.unige.it (F.P.); andrea.calandrino@edu.unige.it (A.C.); lucaramenghi@gaslini.org (L.A.R.); 2 Neonatal Intensive Care Unit, Department of Maternal and Neonatal Health, IRCCS Istituto Giannina Gaslini, 16147 Genoa, Italy; federicamongelli@gaslini.org (F.M.); paolomassirio@gaslini.org (P.M.); 3 Hemostasis and Thrombosis Center, Department of Pediatrics, IRCCS Istituto Giannina Gaslini, 16147 Genoa, Italy; laurabanov@gaslini.org; 4 Pediatric and Neonatal Intensive Care, Department of Emergency Medicine, IRCCS Istituto Giannina Gaslini, 16147 Genoa, Italy; silviaburatti@gaslini.org (S.B.); andreamoscatelli@gaslini.org (A.M.)

**Keywords:** hemophilia A, neonatal bleeding, intracranial hemorrhage, neonatal coagulation, case report

## Abstract

**Introduction**: We report the case of a neonate diagnosed with severe hemophilia A (HA) and conduct a literature review of cases of severe HA presenting at the neonatal age to help define the clinical diagnostic findings and existing differences between the sporadic and familial onset of this condition. **Report of a Case**: A 6-day-old newborn presented with worsening pallor, inappetence, and hyporeactivity for 48 h. The diagnosis was severe hemophilia A (HA), leading to an unfavorable outcome. A literature review focusing on case reports and series focusing on the clinical expression of HA in neonates was conducted, documenting clinical presentation, family history, and outcomes. Literature review: Forty patients were included. HA was observed in five cases (12.5%) of very preterm births (≤32 weeks) and in four cases (10%) of moderately or late preterm births. Seventeen patients (43%) had a family history, with inheritance being sporadic (21 newborns, 53%) or acquired (2 cases, 4%). Clinical onset typically occurred within the first week of life (approximately 8 out of 10 cases), while only three cases (7.5%) had onset after the first month. Inherited cases presented with hemorrhagic states (nine cases), hypovolemic shock (five cases), or intracranial hypertension (two cases). Sporadic cases showed localized bleeding (11 cases), hypovolemic shock (5 cases), or neurological symptoms like seizures and anisocoria (5 cases). Acquired cases included severe intracranial hemorrhage in one case. **Conclusions**: Neonatal HA can manifest with severe symptoms and rapid progression, making early diagnosis crucial. Non-specific signs and the absence of coagulophaty disorders in family history can delay diagnosis. Symptoms like prolonged bleeding, cutaneous hematomas, or intracranial bleeding necessitate ruling out major coagulopathy, and neurological signs require immediate imaging to exclude intracranial bleeding.

## 1. Introduction

Hemophilia type A (HA) is a rare X-linked hemorrhagic disorder characterized by a deficiency in coagulation factor VIII (FVIII). It is the most frequently inherited bleeding disorder diagnosed in neonates, representing approximately 85% of all hemophilic patients [1], with an incidence in males of 1:5000 [2]. The severity of this condition is classified as severe, moderate, or mild according to the FVIII activity level: <1%, 1–5%, and 5–40% of the normal [3].

The consensus among authors about the mode of delivery for mothers expecting a baby known to have or be at risk of HA is avoidance of instrumental delivery, fetal scalp electrodes, and blood sampling, as well as early recourse to cesarean section (CS) as guided by obstetric indications [4]. Approximately one-third of cases have no family history of HA and are referred to as sporadic cases; in severe forms, the proportion of sporadic cases is approximately 55% [5].

Therefore, most cases are not diagnosed in utero and are discovered after a bleeding episode.

The pattern of bleeding observed in neonates with HA is quite different from that typically observed in older children, in which a predominance of muscular and joint bleeding has been reported. In fact, a non-negligible number of cases observed in neonates have an iatrogenic origin, such as venipunctures, heel stab samplings or the administration of intramuscular vitamin K [6], and postsurgical bleeding following circumcision [7].

Interestingly, in a cohort study conducted by Chalmers et al., 2018 [8], in the UK on the frequency of intracranial hemorrhage (ICH) in pediatric patients with inherited coagulation disorders, the overall frequency of primary ICH in 54 newborns with HA was 1.4%.

In addition, a European observational cohort study performed at 12 Hemophilia Centers by Richards et al., 2012 [9], to define the rate of and risk factors for neonatal bleeding, including a total of 508 young patients, found that ICH episodes occurred in 18 babies within the first 28 days of life and were associated with long-term neurological defects in 0.4% of them.

In this regard, although quite rare, the first manifestation of neonatal-onset HA may be acute ICH, which is a potentially life-threatening event for young patients and may represent a diagnostic challenge, especially in this population.

Where treatment is indicated for either the prevention or management of acute bleeding, current practice in developed countries recommends the use of recombinant FVIII concentrates as the treatment of choice for neonates with HA [1]. This is based on the likelihood that these products are associated with the lowest risk of transmitting viral infections rather than concentrated plasma transfusions [10]. Replacement therapy during the neonatal period should be monitored because neonates may require higher doses to achieve the desired factor levels and may demonstrate a shortened factor half-life [11,12].

Currently, gene therapy also offers a potential cure for patients with hemophilia by establishing the continuous endogenous expression of factor VIII or factor IX (FIX) following the transfer of a functional gene to replace the hemophilic patient’s own defective gene [13].

## 2. Case Presentation

Here, we report a clinical case of a newborn with severe HA who presented with some warning signs of coagulopathy in the first days of life, which were unfortunately not promptly identified, leading to a diagnostic delay. Parental consent to publish anonymized clinical data and images of the patient was previously obtained.

A 6-day-old male newborn was admitted to the emergency room with the onset of worsening pallor, inappetence, and hyporeactivity for approximately 18–24 h. He was the first child of healthy unrelated parents with an unremarkable pregnancy history; he was born at term of gestation by dystocic delivery with a vacuum extractor due to prolongation of the expulsive stage and the onset of slight bradycardias during cardiotocographic monitoring; no infective risk factors were reported. Birth weight was 4170 g, cranial circumference (CC) was 36 cm, APGAR score at 1st minute was 9, and APGAR score at the 5th minute was 10. Physical examination at birth was routine, except for the presence of a right parietal cephalohematoma at the site of the vacuum application. Intramuscular vitamin K 1 mg was then administered. After 48 h of life, the general examination was remarkable; in particular, the persistent cephalohematoma did not increase in dimensions or show an altered tactile consistency. The umbilical cord stump did not display any abnormalities, and the site of intramuscular injection and heel prick for neonatal screening (performed immediately after discharge) were neither bleeding nor swollen. A complete blood count was performed before discharge to rule out ongoing bleeding supplying the cephalohematoma, resulting in a hemoglobin (Hb) level of 19 g/dL.

**Figure 1:**
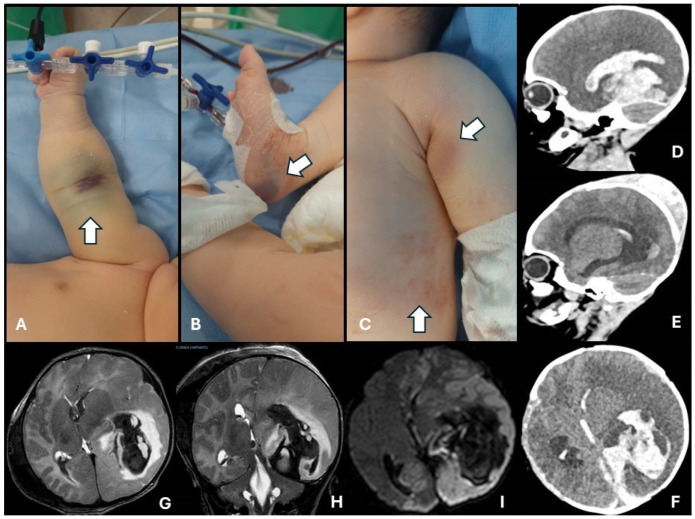
Extensive ecchymosis and edema at venous access sites (panel (**A**), arrow) and at the site of previous capillary sampling (panel (**B**), arrow); hematoma at infant’s holding site level (panel (**C**), arrows). CT scans (panels (**D**–**F**)) and MRI (T1-weighted coronal, panel (**G**); and transversal, panel (**H**), scans) of the extended tetraventicular hydrocephalus, originating from subdural, parenchymal, and intraventricular hemorrhage, with associated ischemic infarction and cytotoxic edema (panel (**I**), DWI-weighted MRI scan), interesting the left hemisphere, with significant compression on the surrounding brain structures.

At the time of ER admission (6th day of life), the patient presented with critical general conditions, characterized by extreme pallor associated with a prolonged capillary refill time (>3 s) and hypotension (arterial pressure 49/44 mmHg); thus, the patient was bradycardic and poorly responsive to intravenous fluid boluses. Extensive ecchymosis and edema at the sites of the previous blood sampling and injection were reported, together with extensive ecchymosis under the armpits, probably a consequence of parental care maneuvers (Figure 1A–C). In addition, the right parietal cephalohematoma was enlarged (CC, 41 cm) and soft to touch. The parents reported that this lesion accretion had begun 48 h after discharge (4th day of life) and gradually continued without causing discomfort to the infant until that moment.

Volumetric support was continued with saline, together with antibiotic therapy with ampicillin and gentamicin, for suspected septic shock. On physical examination, the child displayed hyporeactivity and drowsiness, together with palpebral ptosis of the left eye, fixed mydriasis of the right eye, an absence of photomotor reflex, and generalized hypertone, with the right hand closed into a fist. After blood sampling, prolonged bleeding was observed at the venipuncture site. Laboratory tests showed Hb levels of 6.8 g/dL, a white blood cell count (WBC) of 19,590/mm^3^ with neutrophilic fraction (N) of 14,950/mm^3^, blood glucose levels of 242 mg/dL, and normal renal function, liver enzymes, and C-reactive protein levels. Transfusion of the concentrated erythrocytes was performed in a timely manner, and the patient was intubated and supported with a continuous adrenaline infusion. A total of 1 mg vitamin K IV supplementation was repeated empirically. An extensive coagulation function assessment was performed, which revealed normal PT values (13s) and prolonged PTT (60 s) and low levels of FVIII (0.5%; reference range for our laboratory 65.2–153.4%), leading to the diagnosis of severe HA neonatal onset (FVIII < 1%) according to international guidelines (12, 13). Therapy with FVIII (Elocta 250IU bolus and then continuous infusion of 4 IU/kg/h) was initiated as the results of the blood coagulation tests were available [14,15].

Transfontanellar brain ultrasonography revealed a rightward midline shift resulting from a massive intraventricular and left parenchymal hemorrhage. Given the severity of the intracranial bleeding, additional concentrated red blood cells and concentrated plasma were promptly transfused as a preparation for possible neurosurgical intervention and an urgent CT was performed (Figure 1D–F). It showed tetra-ventricular hydrocephalus associated with extended subdural, parenchymal, and intraventricular cerebral hemorrhage; thus, an ischemic infarction associated with vasogenic edema was found in the left cerebral hemisphere. Subsequently, brain MRI (Figure 1G–I) revealed an extension of the infarction to the entire left hemisphere and the appearance of vascular ischemic infarctions in the frontoparietal contralateral region. During the next few hours, the patient underwent external ventricular derivation. In the following days, clinicians assisted in a progressive worsening of the clinical and neurological conditions, which led to severe hemodynamic insufficiency that was not responsive to resuscitation maneuvers, causing the death of the patient at 13 days of age. Genetic tests were performed on both the newborn and the mother, resulting in the presence of a mutation in exon 14 of the FVIII gene on X chromosome c.4379dup (p.Asn1460Lysfs*2) in the hemizygous. This variant has already been reported to be pathogenic according to the American College of Medical Genetics (ACMG) guidelines. One year later, the parental couple had another female child; this baby was born from normal spontaneous vaginal delivery without any postnatal complications reported. The coagulation screen was regular and FVIII level was 56% immediately after discharge. No bleeding sites nor hematomas were noticed and brain ultrasound assessment was normal, confirming the possible status of severe HA carrier in the newborn.

## 3. Review of the Literature

In the context of the neonatal onset of symptomatology of severe HA, a few cases have been reported in the literature, either sporadic or X-linked inheritance, with variable degrees of severity. All English articles found in the PubMed database by querying MEDLINE with the keywords “hemophilia A”, “neonatal”, and “case report” were revised. We carefully screened 34 articles out of 146 published on this topic over the last 60 years, which reported a total of 40 cases of the neonatal expression of HA. Considering the overall cohort, the disease manifested in patients who were born very preterm (Gestational Age [GA] ≤ 32 weeks) in five cases (12.5%), whereas babies were born at a gestational age of 33–36 weeks comprised five cases (12.5%). All the other reported cases, including the one described here, occurred in patients born at term of gestation, and this may enforce the idea that the occurrence of premature birth is not different in this population compared with the national average [16]. Considering the mode of inheritance, seventeen patients (43%) had a family history (Table 1), whereas in most cases, inheritance was sporadic (21 newborns, 53%) (Table 2) or acquired (2 cases, 4%) (Table 3), which is consistent with previous reports [5].

Among the familiarly inherited diseases, we found a considerable proportion of premature births (four patients, 24%), which was significant compared with the sporadic occurrence group, in which “true prematurity” was reported in one case (5%) and “late prematurity” was present in four cases (19%). Of note, considering the two acquired patients (Table 3), we report that in both cases [50,51] the transplacental transfer of factor VIII:C IgG inhibitor was reported, preventing the disease from causing premature delivery and pregnancy complications.

Regarding the mode of delivery, neonates were born via normal spontaneous vaginal delivery in twenty-five cases (62.5%), dystocic delivery with the use of a vacuum extractor of forceps in four cases (10%), and CS in nine cases (22.5%), corroborating the assumption that the delivery modality does not represent an apparent risk factor for these children [52].

**Figure 2:**
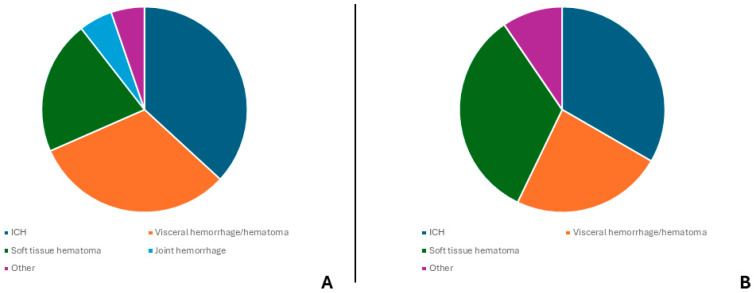
(**A**) Site of the onset of bleeding in family-inherited HA patients. (**B**) Site of onset of bleeding in sporadic HA patients.

In most cases, the clinical onset of symptoms of HA was within the first week of postnatal life (78% of cases), whereas onset occurred after the first month of time in only three cases (7.5%). Clinical onset presented with signs in both the inherited and the acquired condition subcohorts (Table 1 and Table 2): we report two cases (4.8%) where any clinical presentation was absent, twenty cases (47.6%) showing the presence of an active or prior hemorrhagic state in one or more sites without the impairment of general condition, and ten cases (23.8%) where clinical presentation was dramatic, with the presence of severe hypovolemic shock, which required advanced life support. In the remaining seven cases (16.7%), the clinical manifestation involved neurologic (seizures, hyporeactivity, anisocoria) with signs of intracranial hypertension due to the presence of an ICH. Specifical hemorrhage localization is shown in Figure 2A,B.

More specifically, ICH was observed in this retrospective cohort in thirteen cases (28%), with a predominance of subdural hemorrhage (six cases, 46% of all ICHs) and three cases of intraventricular hemorrhage (IVH, 23% of all ICHs). Surprisingly, in the case reported by Koh Kwong Fah et al., 1994 [30], IVH occurred in a term newborn, whereas in the other two cases, the babies were 28 weeks of GA [20,24,25]. Considering the onset time, ICH was mainly reported in the first week of extrauterine life, when it represented the most common onset manifestation (Table 4).

In addition, the extra-axial cranial localization of bleeds (EACH) in the cohort was reported as cephalohematoma or eyelid or palpebral hematoma in six cases (15%), and as a choroidal hemorrhage in one case, accounting for 18% of the total. We acknowledge the predominance of ICH and EACH, occupying 50% of all the reported hemorrhage sites. These data agree with those reported by Kulkarni et al.’s 1999 literature review [53], where major bleeding in the form of ICH and EACH, including sub-galea bleeding and cephalohematoma, was observed in 41% of the collected cases, with an overall predominance of intracranial hemorrhage over all the other described forms.

Regarding the relationship with the mode of delivery, the risk of ICH was highest in those delivered by vacuum extraction, forceps, or CS following labor [48,53].

In this study, we report a new case of the dramatic onset and clinical manifestation of neonatal HA, with subsequent death within the first 2 weeks of life. The prenatal diagnosis of hemophilia is not routinely performed, and screening for this condition is not yet available. Therefore, when a family history is unavailable or insufficiently clarified, as in our case, it is essential to consider HA as a potential differential diagnosis when acute hemorrhage presents as the first clinical sign. Furthermore, it is imperative to emphasize that prompt treatment is crucial in the presence of a reasonable suspicion of bleeding, even prior to radiological confirmation.

## 4. Discussion

The reported case highlights the importance of prompt recognition of the symptoms of hemophilia in neonates, although they can initially be nuanced and subtle. The early identification of these symptoms is critical for timely diagnosis and treatment. In addition to our patient, we reported a similar clinical onset in thirteen other newborns who showed pathological signs during the first physical examination after birth. Unlike this case, most of these patients were diagnosed early and therefore presented favorable outcomes; delayed diagnosis led to psychomotor delays and hemiparesis in only five cases. Among all the reported cases, only three patients died: two from causes independent of HA (one because of bronchopneumonia [18] and the other because of aspiration pneumonia [30] several months after the diagnosis), while only one patient’s death was related to the hemorrhage; in this reported case, the parents refused to administer the life-saving therapy [49]. This finding further underscores the unusual severity and rapid progression of the clinical picture presented by the patient reported in our article in the total absence of risk factors or anamnestic signs suggestive of hemophilia, especially bleeding.

From a genetic point of view, we report that, according to the molecular tests carried out, the mutation discovered in both the child and the mother involved a non-exonic region, confirming the already known assumption that, in cases of severe HA, the most significant part of the pathogenic variants is located in the non-coding areas of the significant FVIII gene, unlike in non-severe forms of the disease [54].

It is essential that neonates presenting with signs of abnormal bleeding are appropriately investigated for the presence of inherited bleeding disorders, including hemophilia. These investigations should include not only baseline coagulation screening tests (PT, aPTT) but also appropriate factor assays [1]. Moreover, it is imperative to rule out the most common vitamin K deficiency-related hemorrhagic status, which may manifest in newborns who do not receive routine perinatal administration [55]. In our case, the normality of PT values guided the differential diagnosis to other, more rare causes of impaired coagulative function, like HA. HA typically results in an isolated prolongation of aPTT; however, although this may suggest a diagnosis of HA, a definitive diagnosis requires measurement of FVIII levels.

We considered the significant advancements in prenatal and postnatal diagnostic techniques over the last few years; therefore, we assume that, in the past, a considerable proportion of cases of neonatal bleeding were not investigated from a genetic perspective and were thus framed as sporadic cases. The same can be said when considering the reported case series’ epidemiology and the frequency of onset manifestations since laboratory tests became as commonly available as they are at present [56,57]. Following the same logic, the definition of ICH as an early-onset symptom of HA should be weighted according to the significant contextual improvements in neuroimaging’s diagnostic capabilities.

We acknowledge that exogenous FVIII therapy for hemophilia A was first introduced in the late 1960s, when cryoprecipitates and plasma-derived concentrates were pioneeringly utilized [58,59], but the introduction of recombinant factor VIII and its subsequent influence on the prognosis and survival rate of these newborns dates to the late 1980s and early 1990s [60], which significantly influences our reasoning.

## 5. Conclusions

The aim of this study was to shed light on this rare condition and identify initial suspicious clinical signs in infants without an early diagnosis of HA, drawing lessons from previous and new clinical cases. We would like to emphasize that when faced with symptoms such as unexplained, out-of-proportion bleeding or hematomas, a neonatologist should always consider a serious entity such as HA and initiate investigations to confirm or exclude the diagnostic hypothesis to allow for timely therapies and ensure a favorable outcome for the newborn. Prompt recognition of these signs is of fundamental importance because the potentially life-threatening consequences of hidden, unrecognized bleeding, such as those in newborns with intracranial hemorrhage, can be avoided.

## Figures and Tables

**Table 1:** Cases of family-acquired HA.

Article Reference	Cases	GA (Week)	Delivery Mode	Sex	BW (g)	APGAR 5′	Genetics	Onset Time (Days)	Clinical Presentation and/or Hemorrhage Site	ICH	Mortality
[17]	1	T	nsVD	M	2920	N.A.	Homozygous Intron 22 inversion in the FVIII gene	3	Macrohematuria	None	+
									Urinary bladder hematoma		
[18]	1	25	nsVD	M	780	N.A.	Hemizygous Intron 22 inversion in the FVIII gene	1	Cephalohematoma	IVH 1	-
									ICH		
[19]	1	T	nsVD	M	N.A.	N.A.	N.A.	13	Hematoma near the joints of both upper arms	None	+
									Articular hematoma		
[20]	2	30	CS	M	710	N.A.	N.A.	1	Venepuncture sites hematomas	None	+
		28	CS	M	1200	N.A.	N.A.	1	ICH	IVH1	+
[21]	1	T	nsVD	M	N.A.	N.A.	N.A.	1	Cephalohematoma; bleeding after circumcision performed at birth	None	+
									Intraosseous skull hematoma		
[22]	1	T	nsVD	M	3500	10	N.A.	2	Hypovolemic shock	None	+
									Retroperitoneal hemorrhage		
[23]	1	T	CS	M	4300	10	Unspecified Intron 1 inversion in the FVIII gene	1	Extended bleeding in the vitamin K injection site	Unspecified	+
									ICH and abdominal hemorrhage		
[24,25]	1	28	CS	F	880	6	Girl with X0	2	Venepuncture sites hematomas	None	+
[26]	1	T	nsVD	M	3450	9	N.A.	3	Hypovolemic shock due to splenic rupture; bilateral subdural hematoma	Subdural	+
[27]	1	T	nsVD	M	3452	10	N.A.	3	Hypovolemic shock due to splenic rupture	None	+
[28]	1	T	nsVD	M	N.A.	N.A.	N.A.	1	Dorsal subcutaneous hematoma	None	+
									Soft tissues hematoma		
[29]	1	T	nsVD	M	3295	10	N.A.	1	Venipuncture sites hematomas; bleeding after circumcision; surgical site bleeding	None	+
[30]	1	T	nsVD	M	3400	N.A.	N.A.	11	Vomiting and stridor due to supraglottic edema with right vocal fold palsy	CBH with midline shift	−
									ICH		
[31]	2	T	DD	M	N.A.	9	N.A.	5	Seizures; left pupil fixed and dilated; left facial droop; anterior fontanel tens	Subdural with midline shift	+
									ICH		
[32]	1	T	nsVD	M	3850	N.A.	Hexon 13 deletion in FVIII gene	2	Hypovolemic shock due to abdominal hemorrhage	None	+
									Splenic hematoma		
[33]	2	T	nsVD	M	3000	10	N.A.	1	Hypovolemic shock due to abdominal hemorrhage	None	+
									Adrenal hemorrhage		

GA, gestational age; T, term gestational age; M, male; F, female; BW, birth weight; ICH, intracranial hemorrhage; nsVD, normal spontaneous vaginal delivery; DD, distocyc delivery; CS, Cesarean section; N.A., not available; IVH, intraventricular hemorrhage; +, alive; −, dead.

**Table 2:** Cases of sporadic HA.

Article Reference	Cases	GA (Week)	Delivery Mode	Pregnancy Notes	Sex	BW (g)	APGAR 5′	Genetics	Onset Time (Days)	Clinical Presentation and/or Hematoma/Hemorrhage Site	ICH	Mortality
[34]	1	T	ED	None	M	2760	10	N.A.	18	Hyporeactivity, pallor, and jaundice	Subdural	+
										ICH		
[35]	1	T	CS	Twin pregnancy	M	2250	10	Unspecified Intron 22 inversion in the FVIII gene	4	Hematomas at the sites of venepuncture; seizures due to ICH and/or cerebral venous sinus thrombosis	Subdural	+
[33]	2	36	ED	None	M	3300	10	N.A.	2	Hypovolemic shock due to hepatic hematoma	None	+
[36]	1	36	CS	Twin pregnancy	M	N.A.	N.A.	N.A.	1	Dorsal cutaneous hematoma	None	+
[37]	3	T	ED	None	M	N.A.	N.A.	N.A.	8	A tumor in the left gluteal area; hematomas at the sites of venipuncture	None	+
		T	ED	None	M	N.A.	N.A.	N.A.	21	Eyelid hematoma and seizures due to ICH	Parenchymal hemorrhage with midline shift	+
		T	ED	None	M	N.A.	N.A.	N.A.	2	hematomas at the sites of venipuncture; cephalohematoma	None	+
[38]	1	T	N.A.	N.A.	M	N.A.	N.A.	N.A.	14	hematomas at the sites of venipuncture; radial artery bleeding pseudoaneurysm	None	+
[31]	2	T	DD	N.A.	M	N.A.	7	N.A.	4	Cephalohematoma; jaundice; seizures due to ICH	Subdural	+
[39]	2	T	ED	None	M	N.A.	10	N.A.	2	Hepatomegaly and hypovolemic shock due to hepatic hematoma	None	+
		T	DD	None	M	3750	9	N.A.	1	Hypovolemic shock due to hepatic hematoma	None	+
[40]	1	T	EU	None	M	3840	10	N.A.	3	Paleness and severe palpebral edema; cephalohematoma; and retro-auricular hematoma	None	+
[41]	1	T	EU	N.A.	N.A.	N.A.	N.A.	N.A.	4	Choroidal hemorrhage with retinal detachment after eye surgery for Peter’s type 2 abnormality	None	+
[42]	1	T	N.A.	None	N.A.	N.A.	N.A.	N.A.	21	Hematomas at the sites of artery puncture; radial artery bleeding pseudoaneurysm	None	+
[43]	1	36	DD	None	M	2700	9	N.A.	1	Seizures; anisocoria due to ICH	Subdural	+
[44]	1	T	ED	None	M	3260	9	N.A.	2	Hematemesis and gut bleeding; hypovolemic shock	None	+
[45]	1	35	ED	GDM	M	2540	N.A.	Unspecified Intron 22 inversion in the FVIII gene	4	Hemopneumothorax after surgical tracheo-oesophageal fistula-oesophageal atresia repair	None	^
[46]	1	36	ED	None	M	2900	9	N.A.	5	Hematomas at the sites of venepuncture; abdominal hematoma due to splenic rupture	None	+
[47]	1	T	ED	None	M	2810	10	N.A.	180	Cutaneous hematoma forearm and iliac crest	None	+
[48]	1	26	CS	Polidramnios, unique umbilical artery	M	635	9	de novo mutation of the FVIII gene (c.4379del1A)	80	None (accidental diagnosis during pre-surgery blood routine)	None	+
[49]	1	T	CS	None	M	3030	10	N.A.	5	Seizures, hypertonia, and hyporeactivity due to ICH	Subdural	−

GA, gestational age; T, term gestational age; BW, birth weight; ICH, intracranial hemorrhage; nsVD, normal spontaneous vaginal delivery; DD, distocyc delivery; CS, Cesarean section; N.A., not available; IVH, intraventricular hemorrhage; +, alive; −, dead.

**Table 3:** Cases of acquired HA.

Article Reference	Cases	GA (Week)	Delivery Mode	Pregnancy Notes	Sex	BW (g)	APGAR 5′	Genetics	Onset Time (Days)	Clinical Presentation and/or Leading Hematoma/Bleeding Site	ICH	Mortality
[50]	1	T	nsVD	Transplacental transfer of an acquired factor VIII: C inhibitor	M	4080	10	Acquired hemophilia due to inhibitor antibodies to factor VIII	5	Hyporeactivity, hypovolemic shock due to ICH	Parenchymal hemorrhage with midline shift	+
[51]	1	T	CS	Transplacental transfer of an acquired factor VIII: C inhibitor	N.A.	2970	10	Acquired hemophilia due to inhibitor antibodies to factor VIII	1	None (prophylactic administration of recombinant FVII in the immediate neonatal period)	None	+

GA, gestational age; T, term gestational age; M, male; BW, birth weight; ICH, intracranial hemorrhage; nsVD, normal spontaneous vaginal delivery; CS, Cesarean section; N.A., not available; +, alive; −, dead.

**Table 4:** ICH as the onset manifestation of HA according to the postnatal life percentiles of the 40 reviewed cases of neonatal HA.

Clinical Onset Time Percentile	Clinical Onset Time (Days)	ICH
<25th	≤1	4 cases (31%)
25th–50th	2–3	1 case (8%)
50th–75th	4–9	5 cases (38%)
>75th	≥10	3 cases (23%)

## Data Availability

The data presented in this study are available on request from the corresponding author due to privacy and ethical restrictions.
